# A suicide prevention strategy for youth presenting to the emergency department with suicide related behaviour: protocol for a randomized controlled trial

**DOI:** 10.1186/s12888-019-2422-y

**Published:** 2020-01-14

**Authors:** Daphne J. Korczak, Yaron Finkelstein, Melanie Barwick, Gloria Chaim, Kristin Cleverley, Joanna Henderson, Suneeta Monga, Myla E. Moretti, Andrew Willan, Peter Szatmari

**Affiliations:** 1 Department of Psychiatry, Hospital for Sick Children, , Toronto, ON Canada; 2 Research Institute, Hospital for Sick Children, Toronto, ON Canada; 3 Department of Psychiatry, Faculty of Medicine, University of Toronto, Toronto, ON Canada; 4 Division of Paediatric Emergency Medicine, Hospital for Sick Children, Toronto, ON Canada; 5 Division of Clinical Pharmacology and Toxicology, Hospital for Sick Children, Toronto, ON Canada; 6 Department of Paediatrics, Faculty of Medicine, University of Toronto, Toronto, Canada; 7 Institute of Clinical Evaluative Sciences, Toronto, ON Canada; 8 Dalla Lana School of Public Health, University of Toronto, Toronto, ON Canada; 9 Lawrence S Bloomberg Faculty of Nursing, University of Toronto, Toronto, ON Canada; 10 Margaret and Wallace McCain Centre for Child, Youth & Family Mental Health, Centre for Addiction and Mental Health, Toronto, ON Canada; 11 Cundill Centre for Child and Youth Depression, Centre for Addiction and Mental Health, Toronto, ON Canada; 12 Clinical Trial Unit, Ontario Child Health Support Unit, Hospital for Sick Children, Toronto, ON Canada; 13 Institute of Health Policy Management and Evaluation, University of Toronto, Toronto, ON Canada

**Keywords:** Suicide prevention, Self-harm, Psychotherapy, Emergency mental health, Child and adolescent psychiatry, Suicide attempt

## Abstract

**Background:**

Suicide is a leading cause of death among adolescents in North America. Youth who present to the Emergency Department (ED) with acute suicidality are at increased risk for eventual death by suicide, thereby presenting an opportunity for secondary prevention of suicide. The current study evaluates the effectiveness of a standardized individual and family-based suicidal behaviour risk reduction intervention targeting adolescents at high-risk for suicide.

**Methods:**

A randomized controlled trial (RCT) will be conducted to evaluate the effectiveness of a manualized youth- and family- based suicide prevention strategy (SPS) as compared with case navigation (NAV) among adolescents aged 12 to 18 years of age who present to the ED with acute suicidal ideation (SI) or suicide risk behaviours (SRB). We will recruit 128 participants and compare psychiatric symptoms including SI/SRB, family communication, and functional impairment at baseline and follow-ups (post-intervention [6 weeks], 24 weeks). The primary outcome is change in suicidal ideation measured with the Suicide Ideation Questionnaire- Junior. SRBs are measured with the Suicide Behaviour Questionnaire. Secondary outcomes are change in depressive and anxious symptoms measured with semi-structured psychiatric interview and Screen for Child Anxiety Related Disorders; acute mental health crises measured by urgent medical (including ED) visits; family communication measured with Conflict Behaviour Questionnaire, functional impairment measured by Columbia Impairment Scale; cost effectiveness, and fidelity of implementation measured by audio recording and fidelity checklist.

**Discussion:**

Results of this study will inform a larger multi-centre RCT that will include both community and academic hospitals in urban and rural settings. Study results will be shared at international psychiatry and emergency medicine meetings, in local rounds, and via publication in academic journals and clinician-oriented newsletters. If effective, the intervention may provide a brief, scalable, and transportable treatment program that may be implemented in a variety of settings, including those in which access to children’s mental health care services is challenging.

**Trial registration:**

ClinicalTrials.gov: NCT03488602, retrospectively registered April 4, 2018.

## Background

Suicide is a global public health problem, claiming more than one million lives each year [1], and is a leading cause of death among youth [2–4]. In Canada, suicide is responsible for one in four deaths among adolescents aged 15 to 19 years, with more youth taking their own lives than the top 10 fatal diseases in this age group combined [2, 3]. Suicide also has profound implications for families and communities and incurs massive societal costs estimated at over 93 billion dollars per year in the United States alone [5]. For these reasons suicide is of considerable global significance, and is a priority area of the World Health Organization [6]. Many advocates argue that truly addressing this massive problem requires that suicide is recognized as fulfilling the criteria for a specific mental disorder [7–9].

Despite the widespread appeal of primary suicide prevention programs, successes have been limited. In fact, studies examining trends have identified increasing suicide rates among females between 1980 and 2008 [3], with both males and females experiencing an increase in the medical severity of their emergency department (ED) presentations for suicide-related behaviours since 2004 [10]. Worldwide, for every suicide death there are 20 to 40 attempts [3, 11]. The majority (90%) of youth who attempt suicide either have an underlying mental illness [12] that is commonly undetected and untreated [12] or are at risk of emergence of a psychiatric disorder in young adulthood (e.g. schizophrenia, depression or bipolar disorder, anxiety disorder or personality disorder). Unlike more violent methods of suicide (e.g., firearms), which are frequently fatal, drug overdose has high survival rates. Two recent studies reported on the associations between self-harm attempts and suicide deaths in the general population [13] and among adolescents (*n* = 20,471) [14]. More than 99.9% of adolescents who presented to the ED with intentional overdose survived to discharge. However, roughly 1 in 100 of them died by suicide, compared to 1 in 3500 matched peers from the general population, within a median interval of 3.0 years [15]. Also, a recent overview of systematic reviews concluded that the ED is likely the most promising site for the introduction of an effective suicide prevention strategy. Taken together, these data confirm that adolescents presenting to the ED following a suicide attempt are at increased risk of suicide, and provide a very promising opportunity for secondary suicide prevention through the use of an effective suicide prevention strategy (SPS).

There is presently an urgent unmet need for effective SPSs for youth who present to the ED with suicidal ideation or behaviour, to prevent suicide and also to reduce suicide risk behaviours (SRBs; non-fatal self-injurious behaviours with suicidal ideation, suicide ideation with intent) which are common and frequently precede suicide attempts [16]. Usual care typically consists of suicide risk assessment followed by recommendations for patient- or family- directed walk-in or community mental health care, and does not include any suicide-specific intervention. Because of the strong association between suicide and mental illness, particularly depression, some researchers have targeted depression (or other underlying mental disorder) among youth in order to reduce suicidality. However, several of these interventions have been ineffective in decreasing SRBs [17]. Other interventions have aimed to increase engagement in aftercare by improving linkages from the ED to community mental health services (case navigation) [18, 19]. These studies have reported that although case navigation increases attendance at ED-aftercare mental health appointment, it does not lead to improvements in clinical or functional outcomes [18, 19]. As such, interventions aimed at improving mental health have not been shown to reduce SRBs for high-risk youth.

The current study is informed by available data examining the effectiveness of direct SPSs among youth [17]. One systematic review (2011) of suicide-prevention intervention studies for youth seeking help for self-harm or suicide noted that of 15 RCT’s, only one study reported effects on SRB, and that overall, methodological weaknesses (e.g. risk of bias, report of outcome data) limit data interpretation and progress in the field [20]. When effective, SPSs involved lengthy (6–12 months) therapies [21, 22] which are expensive and may be impractical in many settings, and report participation rates as low as 50% [22]. Conversely, ultra-brief interventions (e.g. single session or telephone follow-up) have not been effective in decreasing SRBs [18, 23, 24] as they are likely of insufficient duration and depth to yield therapeutic benefit. It was further demonstrated that an effective SPS for adolescents included a family component in order to impact suicidal behaviour [17]. This is consistent with research that has highlighted family conflict as a particularly salient risk factor for SRB among adolescents [25, 26]. In a study comparing 3 months of weekly family-based therapy (FBT) for 66 suicidal adolescents, FBT led to greater reductions in suicidal thinking and depressive symptoms compared with treatment as usual [21]. However, the study had several important weaknesses: assessors were not blinded to intervention status, suicide attempts were not captured, and the sample consisted of predominantly (~ 75%) very low income African-American families; all considerations that may limit validity of the study and its generalizability to other contexts.

The present study addresses both the practical and the methodological limitations of previous research in several ways. It targets the most vulnerable, high-risk adolescents (those with SRB) [13, 14] and incorporates key therapeutic components previously shown to be effective [7, 21, 26]. It optimizes treatment duration and intensity, as an immediate-access, 6-week intervention. In addition, the intervention is evaluated using a rigorous Randomized Controlled Trial (RCT) design, thereby increasing confidence in the study results. Finally, implementation outcomes (fidelity to delivery) are included and will inform how the program may be scaled up in community EDs, which may have more limited access to mental health resources following demonstration of effectiveness. We hypothesize that a youth- and family-focused suicide prevention strategy added to usual care (SPS + UC [*Intervention*]) would lead to decreased SRB at 6-month follow-up as compared with case navigation added to usual care (NAV + UC [*Control]*).

## Study objectives

### Primary objective

To determine the effectiveness of a novel, ED-based, SPS in reducing suicidal ideation and risk behaviours (SRB).

### Secondary objectives

To compare the intervention with a control comparator with respect to effect on (2a) symptoms of mental illness; (2b) acute mental health crises (ED and unscheduled health care visits) (2c) family communication; (2d) functional impairment; (2e) cost effectiveness; and (2f) to examine fidelity to the program determine whether the intervention was delivered as intended.

## Methods

### Study design

**Fig. 1:**
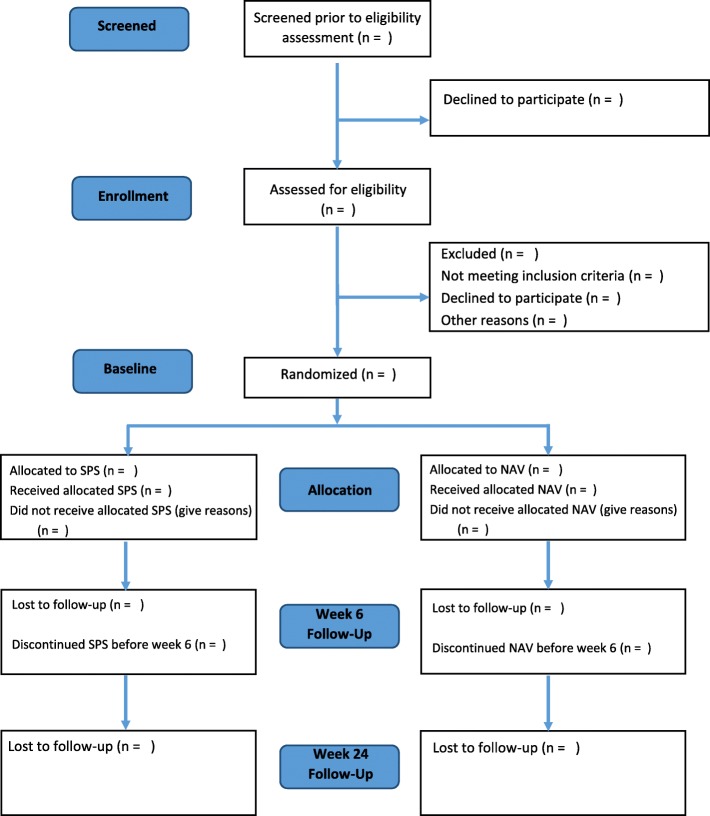
Flow of participants through the study. V = Visit; SPS = Suicide Prevention Strategy; NAV = Case Navigation

**Fig. 2:**
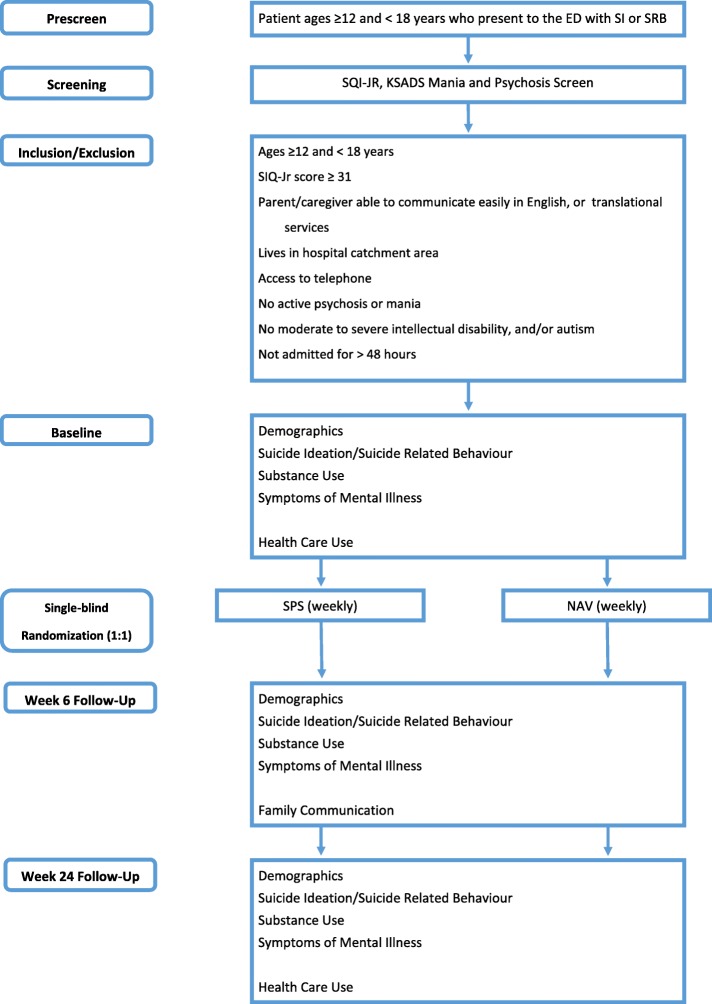
Study protocol: CONSORT diagram for reporting RCTs. V = Visit; SPS = Suicide Prevention Strategy; NAV = Case Navigation

This is a single-blinded, RCT of a SPS + UC (intervention) with NAV + UC (control) in high-risk youth who present to the ED with suicidality using a superiority framework. We will recruit 128 adolescents to be randomized with a 1:1 allocation (64 adolescents per arm). All participants will receive usual care as suggested by the ED clinical team. Overviews of the flow of participants and the study protocol are presented in Figs. 1 and 2, respectively.

### Study setting

The study is conducted at an academic, tertiary care paediatric hospital within the city of Toronto, Canada. The ED is a large, dedicated children’s ED with over 77,000 visits each year. Psychiatric services within the hospital include an Urgent Care Clinic (UCC), outpatient psychiatric care and an inpatient psychiatry unit.

### Participant selection

Consenting youth ages 12 to 18 years who present to the ED with acute suicidal ideation or behaviour (including suicide attempt) will be randomized to intervention or control.

#### Inclusion criteria

1. Ages ≥12 and < 18 years,
2. Presenting to the ED with Suicidal Ideation Questionnaire-Jr [27] (SIQ-Jr) score ≥ 31,
3. Has a participating parent or caregiver who is able to communicate in English or is willing to communicate using a hospital-organized translator,
4. Living in the hospital catchment area,
5. Access to a telephone (mobile or land line).

#### Exclusion criteria

1. Active psychosis or mania (mood elevation score ≥ 3 on the Schedule of Affective Disorders and Schizophrenia for School-Aged Children [28] (KSADS) screen),
2. Moderate to severe intellectual disability, and/or autism based on parent report or clinical chart,
3. Admitted to hospital for self-harm for > 48 h,

### Procedure

#### Recruitment

**Fig. 3:**
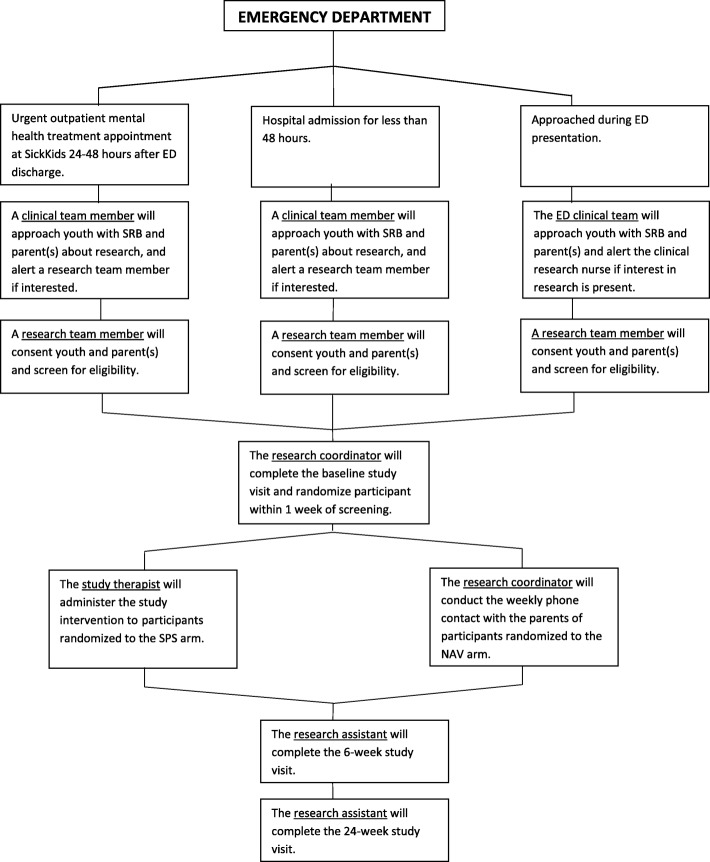
Participant Recruitment and Study Team Assignment

To ensure that all eligible subjects are captured, recruitment will occur in three settings: the ED, the Urgent Psychiatric Care Clinic, and the Psychiatry Inpatient Unit. All subjects will have presented to the ED and meet the inclusion criteria as above, however, some subjects may have missed the research team during their ED visit and may be captured shortly afterward in one of the other two settings. An overview of the recruitment process is presented in Fig. 3.

#### Emergency department (ED)

A research team member in the ED will conduct a pre-screen of patients admitted to the ED using the hospital electronic charting system, identifying patients within the eligible age range (12 to 18 years old) who present with SRB. A clinical team member in the ED will determine the youth’s and parents’ interest in research participation. The eligibility screening assessment will be conducted by a research team member for interested youth and parents/caregivers.

The research staff will review the consent with the youth and the parent(s)/caregiver, and each individual will consent separately for their involvement in the family-based component of the intervention. The youth will also have the opportunity to give permission to access administrative data collected by the Institute for Clinical Evaluative Sciences (ICES), a database in Ontario that stores health patient records, as an optional additional research study component. ICES data will be used to monitor participants’ scheduled and unscheduled medical and hospital visits.

In the event that one of either youth or parent does not consent to the trial it will be considered to be a refusal to participate, and neither youth nor parent/caregiver will be enrolled in the study. Enrolled youth will be referred to the study research coordinator.

#### Urgent care clinic (UCC)

Youth presenting to the ED who are eligible at pre-screen and referred for UCC assessment may be approached during their initial UCC clinic appointment, typically within 24 to 48 h of the index ED visit. A clinical team member who is part of the patient’s care team will ask the youth and parent/caregiver for permission to be approached for research. A research team member will complete the screening and consent.

#### Inpatient unit

Youth presenting to the ED who are eligible at pre-screen and are subsequently admitted to hospital and discharged within 48 h of hospitalization will also be approached for study recruitment. A clinical team member from the inpatient unit will ask the youth for permission to introduce the study. A research team member will complete the screening and consent with the youth and caregiver for those that are interested in study participation.

#### Study visits and intervention

Following informed consent, the study coordinator will contact the participant by telephone within one business day to review the study procedures and book the baseline study assessment visit, to occur within 1 week of the index ED visit. The six-week and 24-week (post-randomization) study visits will also be booked at that time. The baseline study assessment visit will be completed either at the study hospital or at the participant’s home, depending on availability and participant preference. Following completion of the baseline assessment, the participant will be randomized to either the intervention or control arm by the study coordinator.

The study therapist will contact the participants randomized to receive the intervention and schedule the first treatment session. The study coordinator will contact participants randomized to the control arm and initiate the control arm process (NAV) via telephone. In total there are four study visits: screening (the time of presentation in the ED, in-patient or UCC clinic appointment), baseline (within 1 week of screening), 6 weeks post-randomization (post-intervention/control), and 24 weeks post-randomization. Table 1 describes the schedule of administration of study measures.

**Table 1:** Schedule of Assessments

Variable	Instrument	Timeline								
		Baseline	Week 1	Week 2	Week 3	Week 3	Week 4	Week 5	Week 6	Week 24
Baseline Measures										
Psychiatric Symptoms	K-SADS-PL Mania and Psychosis Screening [28] ^a^	x								
	K-SADS-PL Depression Inventory (DEP-C) [28] ^a^	x							x	x
Suicide Related Behaviour	Suicide Behavior Questionnaire –Revised [29] ^a^	x							x	x
Substance Use	Alcohol and Substance Use Questionnaire^a^	x								
Primary Outcome Measure										
Suicidal Ideation	Suicidal Ideation Questionnaire – Jr [27] ^a^	x							x	x
Secondary Outcome Measures										
Symptoms of Mental Illness	Modified Disruptive Behavior Scale (MDBS) Rating Scale [30] ^a^	x							x	x
	Screen for Child Anxiety Related Disorders [31] ^a^	x							x	x
	Strengths and Difficulties Questionnaire (SDQ) [32] ^b^	x							x	x
	Strengths and Weaknesses of ADHD-Symptoms and Normal-Behavior (SWAN) Scale [33] ^a^	x							x	x
Health Care Use	Health Care Utilization Survey (HCUS)^b^	x	x	x	x	x	x	x	x	x
Family Communication	Conflict Behavior Questionnaire [34] ^c^	x							x	x
Impairment	Columbia Impairment Scale [35] ^c^	x							x	x
Covariates										
Demographics Factors	Demographic and Lifestyle Information^c^	x								
Life Stressors	Life Problems Inventory [36] ^a^	x							x	x
Temperament	Children’s Affective Lability Scale [37] ^b^	x							x	x

^a^Child report; ^b^ Parent report; ^c^ Child and parent report

#### Assignment of interventions

##### Treatment allocation and concealment

Participants will be allocated to either the intervention or the control study arms in a 1:1 ratio using a randomization sequence stratified by age and sex. The order of allocation will be randomly generated via REDCap [37] by a research staff member unassociated with the study. The study coordinator will randomize each participant at the end of the baseline study visit by creating a record for the participant in REDCap; the next available allocation can only be accessed at this time.

The study coordinator randomizes the participant and is not blinded to allocation. The coordinator is responsible for communicating with participants, scheduling appointments, and entering outcome measure data as a means of concealing the allocation from the research assistant. The coordinator also instructs the participants not to disclose allocation to the research team member who completes the outcome assessment. A research team member that is blinded to treatment allocation and study hypotheses is responsible for administering outcome measures at 6 and 24-week study assessment visits. We cannot blind the study coordinator, study therapist, or the youth and families to treatment allocation.

##### Intervention

A manualized program that addresses the most common thoughts, feelings and conflicts that suicidal youth experience and teaches coping and safety skills to adolescents and their families. The program will be administered once weekly for 6 weeks by a trained therapist (a certified child and youth counsellor), on an outpatient basis. Sessions consist of one hour spent one-to-one with the youth, followed by one hour with the youth and parents or caregivers.

The therapist provides the youth and parent/caregiver with a manual to use both during and between sessions. The manual is comprised of 6 modules including those centered around safety planning, self-reflection, identifying thoughts and feelings, strengths and coping strategies, family communication, and a final module to review progress.

All study participants also have access to usual care as recommended by the ED clinical team. The intervention will not interfere with the youth’s ongoing plan of care. The study therapist will communicate with the youth’s usual care provider (as identified and permitted by youth participants) at the outset and at completion of the intervention. This communication ensures that the usual care provider is informed about the challenges and strategies for the participant, so that the usual care therapist is better able to assist the participant should similar situations occur in the future.

Intervention sessions will be audio-recorded with youth and the parent(s) or caregiver consent. These sessions will be reviewed by the principal investigator and members of the study team in order to ensure fidelity to the treatment model. The therapist will be supervised by the principal investigator (DK) on a regular basis.

##### Control comparator

The comparator arm consists of weekly telephone calls for up to 6 weeks following the baseline visit. During the telephone call, the study coordinator reviews changes to the youth’s usual care and their contact with community resources in the 7 days prior to the phone call, and provides further information and/or assistance regarding community mental health resources as needed. The aim of the control comparator is to facilitate access to community mental health resources. It provides weekly contact between the study team and participants, as in the intervention arm, however, does not provide therapeutic intervention.

### Measures

Measures utilized in this study elicit information from children and parents using self-report and interviewer-administered measures. Multi-method, multi-informant assessment of psychological symptoms has been shown to increase the accuracy of identifying children with emotional disorders [38].

#### Baseline measures

The Suicide Ideation Questionnaire Junior High School Version (SIQ-Jr) a 15-item measure self-report of suicide ideation (e.g., thoughts about death and dying) that is valid, reliable and sensitive to change [27, 39–43]. Change in SIQ-Jr score is the primary dependent variable. The Suicide Behavior Questionnaire – Revised (SBQ-R) [29] is a 4-item self-report questionnaire that measures suicide behaviour (i.e., previous suicide attempts or the likelihood of future attempts) in the past (baseline) or over the preceding month (follow-up) Screening symptoms of mania, psychosis and assessment of depression will be assessed using the KSADS, Present and Life Version (K-SADS-PL) [28], a semi-structured psychiatric interview that integrates child/adolescent and parent report of current and lifetime history of psychiatric symptoms. The Depression section of the K-SADS- Present Episode Version (K-SADS-Dep C) will be used to assess MDD diagnosis and provide interviewer-rated severity of depressive symptoms at each time point based on integrated child and parent report. Depression symptom severity is rated on a 6-point scale, from none to severe, and has been shown to be a reliable measure of illness severity [44, 45]. The Alcohol and Substance Use Questionnaire is a 9-item measure that is derived from the validated KSADS screen for substance use that has been modified by the addition of items specific to the ED (e.g. “*Did the youth use [the endorsed substance] on the day of presentation to the ED?*”) and assesses youths’ use of alcohol, cannabis, tobacco products, and other substances (see Additional file 1).

#### Secondary outcome measures

To assess symptoms of mental illness from both the child/adolescent and parent perspective, both child/adolescent-report and parent-report will be included. All measures selected have demonstrated reliability and validity among children and adolescents. Measures include: the Modified Disruptive Behavior Scale (MDBS) Rating Scale [30], a 16 item scale that assesses symptoms of externalizing disorders, including oppositionality and symptoms of conduct disorder; the Screen for Child Anxiety Related Disorders (SCARED) [31], a 41-item self-report instrument that assesses generalized, social and separation anxiety symptoms; the Strengths and Difficulties Questionnaire (SDQ) – Parent Report [32], a 25-item parent report of screen of children’s positive and negative attributes; and the Strengths and Weaknesses of ADHD-Symptoms and Normal-Behavior (SWAN) Scale [33], a 30-item instrument that measures symptoms of inattention, hyperactivity and oppositionality. The original scale is a parent report. The self-report version was developed in the Department of Psychiatry at SickKids [46].

The Health Care Utilization Survey (HCUS; see Additional file 1) is an 11-item self- and parent- report questionnaire of participants’ use of available health care services including walk-in, ED, hospital or other health care visits, medication, and the costs or caregiver lost-productivity associated with those services. Changes in the participants’ usual care over the course of the study will be recorded weekly.

Family communication will be assessed using the Conflict Behavior Questionnaire– Child and Parent Versions (CBQ-C) [34], a 20-item children/adolescent- and parent- report measure that examines conflict and communication in the parent-child relationship between specific parent-child dyads (e.g. mother-child, father-child) in the preceding 6 month period (baseline) or 2 week period (follow-up). Family conflict is a hypothesized mediator of the effectiveness of the intervention.

Fidelity of administration of the intervention will be assessed using a fidelity checklist, a 10-item independent rater- and therapist-rated measure developed for the intervention to assess the therapist’s adherence to the manualized intervention. Independent raters review audio-recorded therapy sessions and determine the degree to which 10 key features of the intervention are present and yielding a fidelity score. Therapists are asked to complete an identical measure for participants that refuse to provide consent for audio-recording of therapy sessions. In addition, child/adolescent and parent quantitative and qualitative feedback about the content, delivery and effectiveness of the intervention will be collected using an 8-item feedback form designed for this study (see Additional file 1).

Functional impairment will be measured using the Columbia Impairment Scale – Child and Parent Report (CIS-C) [35]. The CIS is a 13-item instrument that assesses child/adolescent level of function using a 5 point Likert scale, as determined by child/adolescent- and parent- report.

#### Covariates

Child/adolescent demographic and lifestyle information, including age, gender identity and sexual orientation, academic performance, physical activity and social media use, will also be collected at the initial assessment. Parent demographic information, including age, sex, ethnicity, education, occupation, household income, and parent occupation will also be collected at the initial assessment.

Life stress and child/adolescent temperament are both important factors in the lives of children and families that may be extraneous to, but impact upon (i.e. moderate), a participant’s response to the intervention. The Life Problems Inventory (LPI) [36] will be used to examine life stressors and their effects on the participants life, including interpersonal chaos, impulsivity, and emotional dysregulation. Parent report of their child’s ability to regulate their emotions will be assessed using the Children’s Affective Lability Scale (CALS) [37].

### Ethics

The study has been approved by the SickKids Research Ethics Board (December 2017; REB number 1000056892) and enrolled participants will be reviewed by the study Data Safety Monitoring Board (DSMB). This study has also been registered as a clinical trial (ClinicalTrials.gov: NCT03488602, retrospectively registered April 4, 2018); please see the Additional file 1 provided for registration information. Participants will be withdrawn from the study in the following circumstances: withdrawal of informed consent; serious adverse event which, in the opinion of the investigator, indicates that continued participation in the study is not in the best interest of the participant; participant or parent or guardian non-attendance. In the event a subject is withdrawn due to an adverse event, this will be documented and participants will continue to be followed until these events are resolved or stabilized, if possible.

### Patient and public involvement

The intervention was developed in response to patients’ and families’ experiences of mental health care for acute suicidality as being specific to centre, to time/day of presentation, and to assigned therapist. Individual patient and family interviews and therapist focus groups were conducted to inform the design and the delivery of the intervention. Patients and public members were not involved in the design of this RCT protocol, however, youth engagement in other projects at our centre informed the choice of measures, including the CIS. Results will be disseminated to study participants via study newsletters and invitation to attend community mental health meetings in which findings will be shared.

## Data management and analysis

Data will be collected using both self-report and interviewer-administered measures. Where possible, data will be recorded directly into REDCap [47] by participants in order to reduce the potential for missing values, data entry, and encoding errors. All other data will be entered by research staff. Data will be audited by a research team member unaffiliated with the study on a quarterly basis.

### Power analysis

The sample size calculation is based on the assessment of between-group difference in change in SIQ-Jr score. This is a superiority study in which the implementation of the SPS intervention should only be recommended for study in a multi-centre study if the change in the primary outcome is significantly greater than in the control group. A controlled clinical trial of an ED based intervention for suicidal youth has reported effect sizes of 0.4 for suicide attempt and 0.95 for suicidal ideation as measured by the SIQ-JR. A sample size of 128 participants provides 80% power (*p* = 0.05; 2-tailed t-test) to detect an effect size ≥0.5, (based on a standard deviation [SD] of 14 and smallest clinically meaningful difference of 7).

### Data analytic plan

The principle of intent-to-treat will be applied to the analysis of outcomes. The primary outcome, change in SIQ-Jr score, will be compared between arms using repeated measures ANCOVA with the corresponding baseline measure as the covariate. Mean differences and corresponding confidence intervals will be calculated. A two-sided, p-level of 0.05 will be applied. This approach will also be used to compare changes in mental health symptoms, analyzed as continuous outcomes, between arms. The number of unscheduled health care visits will be compared between arms using a Poisson model. The two-sided level for the secondary outcomes will be adjusted (Bonferoni) to account for multiple testing. Implementation outcomes (i.e., fidelity to the intervention protocols) will inform client outcomes. Analysis of implementation data will be descriptive (fidelity to implementation of SPS model), and associative (association of clinician fidelity to intervention with patient outcomes).

### Cost-effectiveness analysis

A cost-effectiveness analysis (CEA) of the incremental costs of the intervention as compared with control in reducing unscheduled health care visits for adolescents presenting with SRB will be conducted. This evaluation will follow the Canadian Agency for Drugs and Technologies in Health guidelines for the conduct of economic evaluations in Canada [48]. Both a health care system and societal perspective will be taken with a six-month time horizon. Direct costs including costs of the intervention, medication and health services use will be collected from study investigators, participants and by linkage with administrative datasets. Indirect costs will include productivity losses of both participants and their caregivers, using the HCUS developed by the team health economist. Cost-effectiveness will be expressed as the incremental cost effectiveness ratio (ICER). Sensitivity analyses will be performed to evaluate the robustness of the results, that is, to determine if the model is sensitive to uncertainty in any specific variable or variables. In addition, probabilistic sensitivity analysis using Monte Carlo simulations will be used to establish a point estimate and the 95% confidence intervals of the ICER.

## Supplementary information

**Additional file 1.** Outcome measures.

## Data Availability

Not Applicable.

## Abbreviations

ED: Emergency Department
NAV: Case Navigation
RCT: Randomized Controlled Trial
SPS: Suicide Prevention Strategy
SRB: Suicide Risk Behaviors
UCC: Urgent Care Clinic
