# Supplementary material for: A suicide prevention strategy for youth presenting to the emergency department with suicide related behaviour: protocol for a randomized controlled trial
Source: BMC Psychiatry. 2020 Jan 14;20:20. doi: 10.1186/s12888-019-2422-y (PMC6961291; doi:10.1186/s12888-019-2422-y)
Supplement: Supplementary file 1 — Additional file 1. Outcome measures. [file 12888_2019_2422_MOESM1_ESM.pdf]

## WHO Data Set

| Data Category | Information |
|---|---|
| Primary registry and trial identifying number | ClinicalTrials.gov:<br>NCT03488602 |
| Date of registration in primary registry | April 4, 2018 |
| Secondary identifying numbers | Not applicable |
| Source(s) of monetary or material support | The Cundill Centre for Child and Youth Depression at the Centre for Addiction and Mental Health (CAMH); The SickKids Foundation; the Centre for Brain and Mental Health at SickKids. |
| Primary sponsor(s) | |
| Secondary sponsor(s) | |
| Contact for public queries | Matthew Tracey, MA – matthew.tracey@sickkids.ca |
| Contact for Scientific queries | Daphne Korczak, MD, MSc<br>The Hospital for Sick Children<br>The University of Toronto |
| Public title | Focused Suicide Prevention Strategy for Youth (FSPS) |
| Scientific title | A Focused Suicide Prevention Strategy for Youth Presenting to the Emergency Department with Suicide Related Behaviour: A Randomized Controlled Trial |
| Countries of recruitment | Canada |
| Health condition(s) or problem(s) studies | Suicidal ideation and behaviour |
| Interventions | Intervention: Manualized individual and family program. Weekly individual and family sessions with a therapist for 6 weeks. |
| | Active comparator: Weekly telephone contact with parents regarding participant health care utilization. Referrals to community mental health resources provided as needed. |
| Key inclusion and exclusion criteria | Ages eligible for study: ≥12 < 18 years<br>Sexes eligible for study: both<br>Accepts healthy volunteers: no |
| | Inclusion criteria: Presenting to the ED with Suicidal Ideation Questionnaire-Jr[24] (SIQ-Jr) score ≥ 31, |
| | Exclusion Criteria: Active psychosis or mania (ie a mood elevation score ≥3 on Kiddie Schedule of Affective Disorders and Schizophrenia[25] (KSADS) screen) |
| Study type | Interventional |
| | Allocation: Randomized controlled trial. Masking: single-blinded (outcome assessor) |
| | Phase: Not applicable |
| Date of first enrolment | March 01, 2018 |
| Target sample size | 128 |
| Recruitment status | Recruiting |
| Primary outcome(s) | Change in suicidal ideation<br>Method of measurement: Suicidal Ideation Questionnaire - Jr<br>Timepoint: Screening, 6 weeks, 24 weeks |
| Key secondary outcomes | Symptoms of mental health, health care use, family communication, impairment. |

## ALCOHOL AND SUBSTANCE USE

**Alcohol**

1. How old were you when you first drank any alcoholic beverage, such as beer, mixed drinks or liquor?

- ☐ Has never drank alcohol. (SKIP to question 2)
- ☐ Has drank alcohol. Age of first use: _______________

1a. How many days in the last month did you drink any amount of alcohol?

__________ days/months

1c. Did you drink on the day of your visit to the ED?

- ☐ Yes
- ☐ No

**Cannabis**

2. How old were you when you used cannabis, including drugs like marijuana, THC, or hash?

- ☐ Has never used cannabis
- ☐ Has used cannabis. Age of first use: _______________

2a. How many days in the last month did you use any amount of cannabis?

__________ days/month

2c. Did you use any cannabis on the day of your visit to the ED?

- ☐ Yes
- ☐ No

**Tobacco**

3. Have you ever smoked cigarettes?

- ☐ Yes
- ☐ No

3a. During the last 30 days (one month), on the days that you smoked, how many cigarettes did you usually smoke?

- ☐ I did not smoke during the last 30 days (one month)
- ☐ Less than 1 cigarette per day
- ☐ 1 cigarette per day
- ☐ 2-5 cigarettes per day
- ☐ 6-10 cigarettes per day

- ☐ 11-20 cigarettes per day
- ☐ More than 20 cigarettes per day

Other Substances

4. Have you ever used any other substances (check all that apply)

- ☐ None
- ☐ Stimulants (*Speed, uppers, amphetamines, dexedrine, diet pills, crystal meth*)
- ☐ Sedatives/Hypnotics/Anxiolytics (*Barbiturates (sedatives, downers), Benzodiazepine, quaalude (ludes), valium, librium, Xanax*)
- ☐ Cocaine (*Coke, crack*)
- ☐ Opioids (*Heroin, morphine, codeine, methadone, demerol, percodan, oxycontin*)
- ☐ PCP (*Angel dust*)
- ☐ Hallucinogens (*Psychedelics, LSD, mescaline, peyote*)
- ☐ Solvents/Inhalants (*Glue, gasoline, chloroform, ether, paint*)
- ☐ Energy Drinks *(Monster Energy, Redbull, Rockstar, etc.)*
- ☐ Caffeine *(Coffee, Iced Coffee, Soft Drinks, etc.)*
- ☐ Other (*Prescription drugs, nitrous oxide, ecstasy, MDA, etc.*)

# Health Care Utilization Survey

Record ID: ______________________

## Extracurricular Activities

Does your child participate in extracurricular activities (outings/sports/hobbies)?

- ◯ No
- ◯ Yes
- ◯ Don't know

If yes, how many hours of extracurricular activities has [Youth] participated in over the last 6 months? (Estimate to best of ability) ______________________

## Academic Activities

Is [YOUTH] currently enrolled in school?

- ◯ Yes
- ◯ No

Reason ______________________

Does [YOUTH] attend full-time (every day) or part time?

- ◯ Full-time
- ◯ Part-time

How many days of school has [YOUTH] missed over the last 6 months ? ______________________

## DOCTOR VISITS

Has [YOUTH] seen a medical doctor in the last month?

- ◯ Yes
- ◯ No

When [YOUTH] attended medical doctor appointments, did someone typically go with [him/her]?

- ◯ Yes
- ◯ No

If yes, who?

- ☐ Mother
- ☐ Father
- ☐ Stepmother
- ☐ Stepfather
- ☐ Brother
- ☐ Sister
- ☐ Other relative
- ☐ Partner (boyfriend/girlfriend)
- ☐ Friend
- ☐ Other

Please specify: ______________________

How does [YOUTH] travel to and from the doctor's office?

- ☐ Personal Vehicle
- ☐ Public Transit
- ☐ Taxi/Uber

If [YOUTH] uses public transit how much do they normally spend traveling to and from the doctor's office? ______________________

If [YOUTH] uses taxi or uber how much do they normally spend traveling to and from the doctor's office? ________________________________

If [YOUTH] or another family member drives, what is the distance to [YOUTHs] doctor's office? ________________________________

## Insurance Coverage

Do you have a drug plan that pays for any of [YOUTH] medications?
- ◯ No
- ◯ Yes
- ◯ Don't know

What is your Drug Plan?
- ◯ employee benefit package
- ◯ Ontario Drug Benefit Program (social assistance)
- ◯ Other
- ◯ Don't know, don't remember
- ◯ Not applicable

Please specify: ________________________________

Do you pay a specific amount before the drug plan begins (in other words, a deductible)?
- ◯ No
- ◯ Yes
- ◯ Don't Know

What is the amount of the deductible? ________________________________

Per:
- ◯ Month
- ◯ Year
- ◯ Don't know what the deductible is

Do you have to pay a certain amount of the total drug price or dispensing fee every time you buy prescription medications (in other words, a co-payment)?
- ◯ No
- ◯ Yes
- ◯ Don't Know

What is the amount of this co-payment? ________________________________

What is the fraction or percentage of prescription medication costs that you pay? ________________________________

Do you have a private health plan that covers other medical expenses such as physical therapy, ambulance services, medical devices etc?
- ◯ No
- ◯ Yes
- ◯ Don't Know

How much do you pay into this plan (the premium)? ________________________________

Time Frame
- ◯ Per Month
- ◯ Per Year

Do you have a private disability insurance?
- ◯ No
- ◯ Yes
- ◯ Don't Know

How much do you pay into this plan (the premium)? ________________________________

Time Frame
- ◯ Per Month
- ◯ Per Year

## Emergency Room Visits

Has [YOUTH] gone to the hospital in an ambulance in the last 6 months?
- ◯ Yes
- ◯ No

How many times did [YOUTH] do so? ____________________

When [YOUTH] went to the hospital in an ambulance, did someone typically go with [him/her]?
- ◯ Yes
- ◯ No

If yes, who?
- ☐ Mother
- ☐ Father
- ☐ Stepmother
- ☐ Stepfather
- ☐ Brother
- ☐ Sister
- ☐ Other relative
- ☐ Partner (boyfriend/girlfriend)
- ☐ Friend
- ☐ Other

Please specify: ____________________

Did you have to pay for the ambulance services?
- ◯ Yes
- ◯ No
- ◯ Don't know/can't remember
- ◯ Not applicable

How much did you spend on these ambulance services? ____________________

Has [YOUTH] gone to the emergency room by some other method of transportation in the last 6 months?
- ◯ Yes
- ◯ No

How many times did [YOUTH] do so? ____________________

Please describe the reasons [YOUTH] went to the emergency room

____________________

## Hospital Admissions

Has [YOUTH] been admitted to the hospital in the last 6 months?
- ◯ Yes
- ◯ No

How many times was [YOUTH] admitted to the hospital? ____________________

When [YOUTH] was admitted to the hospital, did someone typically visit you [him/her]?
- ◯ Yes
- ◯ No

If yes, who?
- ☐ Mother
- ☐ Father
- ☐ Stepmother
- ☐ Stepfather
- ☐ Brother
- ☐ Sister
- ☐ Other relative
- ☐ Partner (boyfriend/girlfriend)
- ☐ Friend
- ☐ Other (please specify)

Please describe the dates and reasons for these admissions to the hospital:

Date of admission ________________________

Reason for being admitted ________________________

Length of time in hospital (days) ________________________

Date of admission ________________________

Reason for being admitted ________________________

Length of time in hospital (days) ________________________

## Allied Health Professionals and Social Service Providers

Have [YOUTH] made visits to health care or social service providers (physiotherapist, social worker, adolescent/school counsellor, children's aid, family counsellor, occupational therapist, psychologists, nurse, chiropractor, police officer, parole officer, support group) in the last 6 months?

- ◯ Yes
- ◯ No

When [YOUTH] made these visits, did someone typically go with [him/her]?

- ◯ Yes
- ◯ No

If yes, who?

- ☐ Mother
- ☐ Father
- ☐ Stepmother
- ☐ Stepfather
- ☐ Brother
- ☐ Sister
- ☐ Other relative
- ☐ Partner (boyfriend/girlfriend)
- ☐ Friend
- ☐ Other (please specify)

Please specify: ________________________

Can you describe who [YOUTH] went to see, how many visits there were, how much was spent on each of these visits and whether you had to pay for this visit yourself or if it was covered by insurance?

Health Professional

- ◯ Family Counsellor
- ◯ Adolescent/School Counsellor
- ◯ Social Worker
- ◯ Psychologist
- ◯ Nurse
- ◯ Other

Please specify: ________________________

Number of Visits ________________________

Amount Spent ________________________

Self-Paid or Insurance

- ◯ Self-paid
- ◯ Insurance

Mileage/Parking ________________________

Health Professional
- ◯ Family Counsellor
- ◯ Adolescent/School Counsellor
- ◯ Social Worker
- ◯ Psychologist
- ◯ Nurse
- ◯ Other

Please specify: ______________________________

Number of Visits ______________________________

Amount Spent ______________________________

Self-Paid or Insurance
- ◯ Self-paid
- ◯ Insurance

Mileage/Parking ______________________________

Health Professional
- ◯ Family Counsellor
- ◯ Adolescent/School Counsellor
- ◯ Social Worker
- ◯ Psychologist
- ◯ Nurse
- ◯ Other

Please specify: ______________________________

Number of Visits ______________________________

Amount Spent ______________________________

Self-Paid or Insurance
- ◯ Self-paid
- ◯ Insurance

Mileage/Parking ______________________________

---

## Loss of time from work (paid or unpaid)

Do you work in paid employment?
- ◯ Yes
- ◯ No

Do you participate in any volunteer activities or unpaid employment?
- ◯ Yes
- ◯ No

Have you had to miss any time from work/volunteer activities to go to the doctor, emergency room or while your child was admitted to the hospital?
- ◯ Yes
- ◯ No
- ◯ Not applicable

Can you estimate how many days, in total, you had to take off? ______________________________

When [YOUTH] went to the doctor, emergency room or were admitted to the hospital, has anyone else (ie. another caregiver) had to miss time from paid employment to help you care for [YOUTH] or accompany [YOUTH]?
- ◯ Yes
- ◯ No
- ◯ Not applicable

Can you estimate how many days, in total, they had to take off? ______________________________

| | |
|---|---|
| During the XXX weeks, were you or other family members prevented from engaging in any activities such as shopping volunteer work, visiting friends, going to the movies etc., to care for [youth] | ◯ Yes<br>◯ No<br>◯ Not applicable |
| Can you estimate how many days (or hours), in total, this was? | ______________________ |
| During the XXX weeks, were you or other family members prevented from engaging in your regular homemaking tasks to care for [youth] | ◯ Yes<br>◯ No<br>◯ Not applicable |
| Can you estimate how many days (or hours), in total, this was? | ______________________ |
| Do you pay for any individual to assist you with homemaking activities? | ◯ Yes<br>◯ No<br>◯ Not applicable |
| Can you estimate how much you spent in the last XXX weeks? | ______________________ |

**A Focused Suicide Prevention Strategy for Youth Presenting to the Emergency Department with Suicide Related Behaviour: A Randomized Controlled Trial**

**Therapy Fidelity Checklist**

**Participant ID:** **Provider:**

**Session:** **Date:**

| Item | Rating |
|---|---|
| Referred to the SAFETY manual | ☐ Yes ☐ No |
| Conducted the correct module for the session (e.g., Module 1 in session 1) | ☐ Yes ☐ No |
| Reviewed the safety plan with the youth | ☐ Yes ☐ No |
| Reviewed what strategies from the preceding session that the youth used over the week | ☐ Yes ☐ No |
| Incorporated previous content (e.g., motivations, goals, coping strategies) in session ("remember when we talked about…") | ☐ Yes ☐ No |
| Brought the parents/caregiver into the session | ☐ Yes ☐ No |
| Reviewed the last week with parent/caregiver | ☐ Yes ☐ No |
| Set an agenda with family at the beginning of session | ☐ Yes ☐ No |
| Addressed communication between youth and parent/caregiver | ☐ Yes ☐ No |
| Assigned tasks for youth and parent/caregiver between sessions | ☐ Yes ☐ No |
